# Cancer-Associated Fibroblasts in a 3D Engineered Tissue Model Induce Tumor-like Matrix Stiffening and EMT Transition

**DOI:** 10.3390/cancers14153810

**Published:** 2022-08-05

**Authors:** Martial Millet, Enola Bollmann, Cassandra Ringuette Goulet, Geneviève Bernard, Stéphane Chabaud, Marc-Étienne Huot, Frédéric Pouliot, Stéphane Bolduc, François Bordeleau

**Affiliations:** 1 CHU de Québec-Université Laval Research Center (Oncology Division) and Université Laval Cancer Research Center, Quebec City, QC G1R 3S3, Canada; 2 CHU de Québec-Université Laval Research Center (Regenerative Medicine Division), Quebec City, QC G1V 4G2, Canada; 3 Centre de Recherche en Organogénèse Expérimentale/LOEX, Université Laval, Quebec City, QC G1J 1Z4, Canada; 4 Department of Molecular Biology, Medical Biochemistry and Pathology, Université Laval, Quebec City, QC G1V 0A6, Canada; 5 Department of Surgery, Université Laval, Quebec City, QC G1V 0A6, Canada

**Keywords:** 3D tumor models, engineered tumor microenvironment, matrix stiffness, ECM remodeling, cancer-associated fibroblasts, EMT, cell contractility, mechanotransduction, bladder cancer

## Abstract

**Simple Summary:**

The physical properties of a tumor, such as stiffness, are important drivers of tumor progression. However, current in vitro tumor models fail to recapitulate the full range of physical properties observed in solid tumors. Here, we proposed a 3D self-assembly engineered bladder model using cancer-associated fibroblasts in which stromal cells produce their extracellular matrix. We then proceeded to assess how our model recapitulates biological and mechanical features found in tumors. We confirmed that stroma assembled by cancer-associated fibroblasts have increased extracellular matrix content and display increased remodeling and higher stiffness. Moreover, normal urothelial cells seeded on the tumor model displayed a mechanotransduction response, increased cell proliferation, cell infiltration within stroma, and displayed features of the epithelial-to-mesenchymal transition. Altogether, we demonstrated that our cancer-associated fibroblast-derived tumor stroma recapitulates several biological and physical features expected from a tumor-like environment and, thus, provides the basis for more accurate cancer models.

**Abstract:**

A tumor microenvironment is characterized by its altered mechanical properties. However, most models remain unable to faithfully recreate the mechanical properties of a tumor. Engineered models based on the self-assembly method have the potential to better recapitulate the stroma architecture and composition. Here, we used the self-assembly method based on a bladder tissue model to engineer a tumor-like environment. The tissue-engineered tumor models were reconstituted from stroma-derived healthy primary fibroblasts (HFs) induced into cancer-associated fibroblast cells (iCAFs) along with an urothelium overlay. The iCAFs-derived extracellular matrix (ECM) composition was found to be stiffer, with increased ECM deposition and remodeling. The urothelial cells overlaid on the iCAFs-derived ECM were more contractile, as measured by quantitative polarization microscopy, and displayed increased YAP nuclear translocation. We further showed that the proliferation and expression of epithelial-to-mesenchymal transition (EMT) marker in the urothelial cells correlate with the increased stiffness of the iCAFs-derived ECM. Our data showed an increased expression of EMT markers within the urothelium on the iCAFs-derived ECM. Together, our results demonstrate that our tissue-engineered tumor model can achieve stiffness levels comparable to that of a bladder tumor, while triggering a tumor-like response from the urothelium.

## 1. Introduction

Solid tumor microenvironment is characterized by biophysical changes in the extracellular matrix (ECM). Notably, a higher deposition and crosslink of ECM proteins, such as collagen and fibronectin, lead to an increased tissue stiffness [1,2]. Those changes are known to affect cell behavior, including invasion, cell proliferation, epithelial-to-mesenchymal transition (EMT), and drug resistance [3,4,5,6]. In addition, there are several cell types involved in tumor progression, ranging from fibroblasts to epithelial cells [7]. Because of this complexity, current models often focus on a limited number of parameters. For instance, while 2D polyacrylamide gels have been useful to recapitulate the stiffness of the tumor, they lack the 3D architecture [8,9,10]. In contrast, hydrogels do provide a complex 3D architecture, but collagen hydrogels cannot achieve high enough stiffness while synthetic biomaterials do not reproduce the fibrillar architecture of the tissue [11,12]. Therefore, we cannot readily recapitulate the complexity, heterogeneity, and physical properties of the tumor microenvironment using current approaches. This underlines the importance of developing new engineered models that combine both stiffness and architecture.

Approaches used in regenerative medicine can recapitulate functional engineered tissues with properties close to their in vivo counterparts [13]. One method to produce functional tissues is the self-assembly technique, which has been used to produce skin, cornea, or cardiac valves that are used in a clinical setting [14,15,16]. With this approach, several teams, including our own, have successfully reproduced both the architecture and mechanical properties of normal tissues, ranging from skin to bladder [17,18]. We have shown previously that we can use tissue-engineering-based self-assembly as a technical approach to produce a tumor supporting stroma [19]. However, the reconstituted stroma of these tissues in these studies is still equivalent to a normal tissue, and far from the expected properties of a tumor stroma. Given that stromal stiffness is a critical driver of tumor progression, improving our ability to modulate the stroma architecture and stiffness of self-assembled tissues would lead to more relevant tumor mimetic models [1].

The principal cells that drive the evolution of the tumor ECM are the cancer-associated fibroblasts (CAFs), which arise from the activation of healthy fibroblasts by cancer cells [20]. CAFs have a pro-tumorigenic role as they are responsible for the increased ECM deposition as well as for the secretion of growth factors, cytokines, chemokines, ECM crosslinking enzymes and degradation proteins such as metalloproteinases (MMPs) [21,22]. Moreover, CAFs are more contractile and can readily remodel collagen and fibronectin [20]. Overall, the increased deposition of the various ECM molecules, release of ECM-remodeling enzymes, and direct remodeling of the ECM by the CAFs, modulate the tumor ECM architecture and composition while increasing its stiffness [23,24]. Numerous 3D models using spheroids, 3D collagen gels, or microfluidic devices have incorporated CAFs to study tumor-related CAFs’ biological features [25,26]. For instance, studies using 3D hydrogels have highlighted how CAFs’ secretome contributes to tumor cell EMT and invasion [26,27,28,29,30], highlighting the importance of including CAFs in 3D models. Considering that the use of healthy fibroblasts allows the self-assembly of a a construct that reproduce a physiologically relevant stroma [19], and given that we have previously shown that we can readily induce primary bladder fibroblasts into iCAFs [31], we hypothesized that the use of iCAFs would allow us to recreate a tumor-like microenvironment.

In this study, we investigated whether we could leverage CAFs within an established 3D self-assembled bladder model to achieve tumor-like mechanical properties, and how the altered stroma influences the response of a normal urothelium. Specifically, we constructed two distinct assemblies of three stromal sheets obtained from normal healthy fibroblasts (HFs) and induced cancer-associated fibroblasts (iCAFs) coupled with urothelial cells to obtain a complete engineered tissue. The ECM content and organization were quantified using immunofluorescence and histochemistry. In addition, we assessed cell contractility within the urothelium using quantitative polarization microscopy (QPOL) and its associated mechanotransduction responses. Our results show an increased collagen content and ECM reorganization in the iCAFs constructs, in line with increased stroma stiffness, which in turn correlates with increased urothelial cell contractility, proliferation, expression of EMT markers, and stromal infiltration of urothelial cells.

## 2. Materials and Methods

### 2.1. Antibodies and Reagents

Primary antibodies used were as follows: Rabbit anti-YAP1 (#14074, Cell signaling Technology, Whitby, ON, Canada); Mouse anti-vimentin (#550513), Mouse anti-E-Cadherin (#610181) and Mouse anti-Ki67 (#556003) were from BD BIOSCIENCES (Franklin Lakes, NJ, USA); Rabbit anti-ZEB1 (#ABD53), Rabbit anti-total Fibronectin (#F3648), Mouse anti-laminin-V (#MAB19562), and Mouse pan-anti-Cytokeratin AE1/AE3 (#MAB3412) were from MilliporeSigma (Billerica, MA, USA); Rabbit anti-vimentin (#ab45939), Rabbit anti-MMP9 (#ab76003) were from Abcam (San Francisco, CA, USA). Secondary antibodies used were as follows: Goat anti-Mouse Alexa Fluor 488 (#A11001), Goat anti-Rabbit Alexa-Fluor 488 (#A11029), Goat anti-Rabbit Alexa-Fluor 594 (#A11037), and Goat anti-Mouse Alexa-Fluor 594 (#A11032) were from Thermo Fisher Scientific. All other chemicals were from MilliporeSigma unless specified.

### 2.2. Cell Isolation and Culture

Healthy bladder fibroblasts (HFs) (FHu5 and FHu6) and healthy dermal fibroblasts (F34) were respectively obtained from bladder diverticulum, neurogenic bladder, and skin biopsies. Urothelial cells (uroHu1) were obtained from a pelvis biopsy. Patient derived cells were isolated as previously described [32]. Briefly, the stroma was separated from the epithelium after an incubation overnight at 4 °C in HEPES buffer with 500 μg/mL thermolysin (Sigma-Aldrich, Saint-Louis, MO, USA). The FHu5, FHu6, and F34 fibroblasts were enzymatically dissociated from the ECM by treating the stroma with 0.125 U/mL collagenase H (Roche, Missisauga, ON, Canada) for 3 h at 37 °C under gentle agitation. Then, fibroblasts were cultured in Dulbecco-Vogt modified Eagle’s media (DMEM) supplemented with 10% fetal bovine serum (FBS) (Invitrogen, Burlington, Canada) and antibiotics (100 U/mL penicillin and 25 μg/mL gentamicin). UroHu1 cells were isolated from the urothelium using trypsin for 30 min at 37 °C and then cultured in DMEM/Ham F12 (DH; ratio 3:1; Invitrogen, Oakville, ON, Canada) supplemented with 5% Cytiva HyClone^TM^ FBS (Fisher Scientific, Ottawa, ON, Canada), 5 µg/mL insulin (Sigma), 0.4 mg/mL hydrocortisone (Calbiochem, San Diego, CA, USA), 0.212 µg/mL isoproterenol (Sandoz, Boucherville, QC, Canada), 10 ng/mL epidermal growth factor (Austral Biologicals, San Ramon, CA, USA), 100 U/mL penicillin, 25 mg/mL gentamicin, and buffered with sodium bicarbonate. The T24 bladder cancer cell line was obtained from the ATCC (HTB-4™) and cultured in DMEM containing 10% FBS and antibiotics. Cells were maintained at 37 °C under an 8% CO_2_ atmosphere and kept for a maximum of 5 passages and were routinely tested for mycoplasma contamination. The cells were authenticated under short tandem repeat analysis by the cell bank.

### 2.3. CAF Induction and Tissue Construction

Conditioned medium (CM) was obtained from confluent T24 bladder cancer cells cultured in 75 cm^2^ flasks. 8 mL of the culture medium was collected and centrifuged at 300 g for 10 min. 7.5 mL of the supernatant was then recovered and supplemented with 0.5 mL FBS and 50 µg/mL ascorbate.

The self-assembly technique was used as previously described to produce a 3D vesical model [33] with the following modifications: the healthy bladder fibroblasts were seeded at a concentration of 3.6 × 10^5^ cells, mixed with 4 × 10^4^ healthy dermal fibroblasts and seeded in culture dishes containing a paper anchorage device weighted by a metal air/liquid support. The cells were then cultured with either DMEM 10% FBS supplemented with 50 µg/mL ascorbate (HFs-stroma sheets) or CM (CAFs-stroma sheets) for 28 days until their neosynthesized ECM proteins were sufficiently assembled to form a sheet. Three stroma sheets were stacked in 60 mm Petri dishes to form two different combinations: 3 HF-stroma sheets (HFs construct) and 3 CAFs-stroma sheets (iCAFs construct). Each 3-sheet stack was clipped at the level of their paper anchorage using ligaclips and put under mechanical load using metal ingots and cultured for four more days to allow their fusion. Constructs were protected from direct contact with ingots by surgical sponge (Merocel). Merocel sponges and ingots were removed the day after, and fusion of the sheets was allowed for 3 additional days in DMEM 10% FCS supplemented with 50 µg/mL ascorbate. The constructs were seeded with 2 × 10^5^ urothelial cells then maintained in submerged condition for one week in DH medium supplemented with 50 µg/mL ascorbate. Then, the constructs were raised at the air/liquid interface using the support device and cultures were pursued for another 3 weeks until complete maturation of the urothelium.

### 2.4. MMPs Activity Assay

The MMPs activity was determined using the MMP Activity Assay Kit (Abcam, San Francisco, CA, USA) according to the manufacturer’s protocol. Briefly, HFs and iCAFs cells were seeded at 5 × 10^4^ cells/well into 12-well plates and allowed to adhere overnight. The conditioned medium was mixed with 50 µL of 2 mM APMA working solution and incubated for 15 min at room temperature followed by the addition of 100 µL of the fluorogenic peptide substrate solution. The fluorescence signal was read after 60 min using the Varioskan Flash microplate reader (Thermo Electron Corporation, Waltham, MA, USA).

### 2.5. Mechanical Testing

Mechanical characterization of the reconstructed stroma was performed under tensile load following an approach described before [34]. Only freshly fused tissue constructs with no urothelium were used for the mechanical tests. Tensile dog bone punches were taken from each tissue sample and the thickness of each sample was assessed using pictures from stained tissue cross-sections and measured in ImageJ. Each tensile sample was tested to failure at a strain rate of 0.2 mm/s on a Instron E-1000 (Instron, Norwood, MA, USA) equipped with a ±10 N load cell. The resulting load data were analyzed for the tensile modulus by least squares regression using an elastic model over the toe region of the curve [35]. The provided tissues Young’s moduli are for a 5% strain deformation.

### 2.6. Immunofluorescence

3D vesical models were snap-frozen in OCT and cut into 7 μm sections using a Leica cryostat. Methanol-fixed cross-sections were labeled with anti-AE1/AE3 (1/1000), anti-vimentin (1/1000, #ab45939), anti-laminin-V (1/400), or anti-Ki67 (1/500) primary antibodies diluted in 1% *w/v* bovine serum albumin (BSA; BioBasic, Markham, ON, Canada) in phosphate buffered saline (PBS) for 60 min at room temperature, rinsed in PBS, and then incubated for 45 min with the anti-Mouse Alexa Fluor 488 or anti-Rabbit Alexa Fluor 594 secondary antibody (1/1000). Negative controls were performed by incubating cross-sections with an IgG1 isotypic antibody (Dako, Burlington, ON, Canada). Nuclei were stained with Hoechst 33258 (1/1000, #H3569, Invitrogen)

Alternatively, the cryo-sections were fixed with 4% paraformaldehyde, permeabilized with 1X PBS 0.1% triton solution, and then stained with an anti-YAP1 (1/50), anti-vimentin (1/100, #550513), anti-ZEB1 (1/50), anti-Fibronectin-Total (1/50) diluted in 1X PBS in 5% goat serum for 2 h in a humid chamber at 37 °C. An anti-rabbit Alexa-Fluor 488 was then incubated at 1/200 dilution for 2 h in a humid chamber at 37 °C. Nuclei were stained with DAPI (4′,6-Diamidino-2-Phenylindole, Dilactate, #D3571, ThermoFisher). Samples were then mounted using Fluoromount-G (#0100-01, Southern Biotechnology, Birmingham, AL, USA). Fluorescent images were acquired using a Zeiss LSM 900 confocal microscope equipped with a 40x objective or a Zeiss Axio Imager M2 microscope equipped with an AxioCam HR Rev3 camera (Carl Zeiss Microscopy, Thornwood, NY, USA).

### 2.7. Image Quantification

Quantification of the collagen content from color images of the Picrosirius staining was performed with Fiji (Fiji Is Just ImageJ, open source image processing package). The white balance of each color image was corrected post-acquisition using an ImageJ macro (White balance correction_1.0). RGB images were then converted to Luminescence and an automatic threshold was set using the Huang algorithm to exclude the cell-specific signal. The area of the identified region of interest corresponding to the Picrosirius-stained collagen was measured and compared to the area of the whole stroma visible in the image.

Quantification of the confocal images was performed using the Fiji. Briefly, a maximum z-projection was obtained from the image stacks, subjected to a background subtraction (rolling ball; 50 pixels radius) and a mean filter (1 pixel radius). For FN quantification, the mean signal was measured over the full images. For YAP and ZEB1 quantification, segmented lines of width close to the average width of the nuclei (10 μm) were used to quantify the signal in nuclei. Using the DAPI channel, nuclei positions were determined to measure the associated YAP and ZEB1 signals. For vimentin quantification, a segmented line of defined length and width (35 μm) was used to quantify the signal of vimentin according to the distance from the urothelium–stroma interface. Considering the increased confluence of cells in urothelium, the DAPI channel was used to define the nucleus. The vimentin fluorescence signal was normalized to the signal at the urothelium–stroma interface for all conditions using Prism (GraphPad Software).

### 2.8. Gelatinase Activity Assay

HFs and iCAFs were seeded at a density of 5 × 10^3^ cells/well on 8-well Millicell EZ slides (Millipore, Burlington, MA, USA), and were subjected to overnight incubation with 100 mg/mL of highly quenched fluorescein-labeled gelatin (DQTM Gelatin D-12054, Molecular ProbesH, Eugene, Oregon). The cells were then washed with PBS and fixed with 4% *v/v* paraformaldehyde/PBS for 30 min followed by permeabilization with 0.25% *v/v* Triton X-100/PBS for 30 min, blocked with 5% BSA in PBS for 1 h and then stained. The cells were incubated with a rabbit anti-MMP9 (1/200) at room temperature for 1 h in PBS 5% BSA, rinsed in PBS before incubation with the Alexa Fluor 488 conjugated anti-rabbit secondary antibody (1/1000) in PBS for 1 h. Cells were then mounted and counterstained using the VECTASHIELD mounting medium with DAPI (Vector Laboratories, Peterborough, UK) and imaged on the Zeiss Axio Imager M2 microscope.

### 2.9. Quantitative Polarization (QPOL)

Microscopy cross-sections of urothelial cells in a 3D vesical model colored with the Picrosirius Red stain and stain-free microscopy cryosections of these 3D vesical model samples were processed via QPOL microscopy technique. The polarized signal was then imaged and quantified using both white and monochromatic red lights in QPOL as previously described [36,37]. Briefly, QPOL imaging was performed using an Axio Vert microscope (Zeiss, Toronto, ON, Canada) equipped with 10 × 0.35 N.A. and 20 × 0.5 N.A. polarization objectives, and an Axiocam 305 monochromatic camera (Zeiss). The microscope is composed of a motorized rotating linear polarizer (max speed of 20° s^−1^; Thorlabs, Newton, NJ, USA) positioned directly under the illumination source and above the condenser, and a circular analyzer [37]. The Zen lite software was used for image acquisition (Zeiss). For stained samples and strain-free samples, an image sequence was acquired using the Axiocam 305 at each 10° step of the rotating polarizer over a 0–180° range. The sequence of images was then processed with a MATLAB code in order to obtain a pixel-by-pixel retardance image, from which the area of the collagen regions was extracted and analyzed using the OrientationJ ImageJ plugin.

### 2.10. RT-qPCR

The RNA was extracted using the EZ-10 Spin Column Total RNA Miniprep Super Kit (BioBasic, Markham, ON, Canada). The RNA quality was assessed on a bioanalyzer using the Agilent RNA 600 Nano kit. 1 µg of RNA was used to reverse transcribe into cDNA using the High-Capacity cDNA Reverse Transcription Kit (Applied Biosystems, Foster City, CA, USA). qPCR was performed using the DyNAmo HS SYBR Green qPCR kit (ThermoFisher, Waltham, MA, USA), following the manufacturer’s instructions. The primers used to quantify mRNA of COL1A1, FN1, MMP2, and MMP9 are as listed in Supplementary Table S1. The gene expression was normalized with the β2-microglobulin transcript.

### 2.11. Collagen Gel Contraction Assay

Collagen gels were prepared with the PureCol^®^ EZ Gel solution (Sigma-Aldrich, Saint-Louis, MO) in DMEM 20% FBS supplemented with 100 IU/mL penicillin and 25 μg/mL gentamicin, as well as 2.5 × 10^3^ iCAFs. The mixture (500 μL) was cast into a 24-well culture plate and allowed to polymerize at 37 °C for 90 min. After polymerization, the gels were gently released from the plate with a blade. After 24 h, the surface area of the gels was measured with the ImageJ software (NIH, Bethesda, MD, USA). The values were normalized to control gels without cells and the % of initial area was calculated.

### 2.12. Statistical Analysis

GraphPad Prism was used for the graphical representation of data and statistical analyses. The results are expressed as mean ± standard error. Differences between the groups were considered significant at *p* < 0.05. Data were interpreted using an unpaired *t*-test.

## 3. Results

### 3.1. iCAFs-Derived Tissues Display Tumor-like Composition and Properties

**Figure 1:**
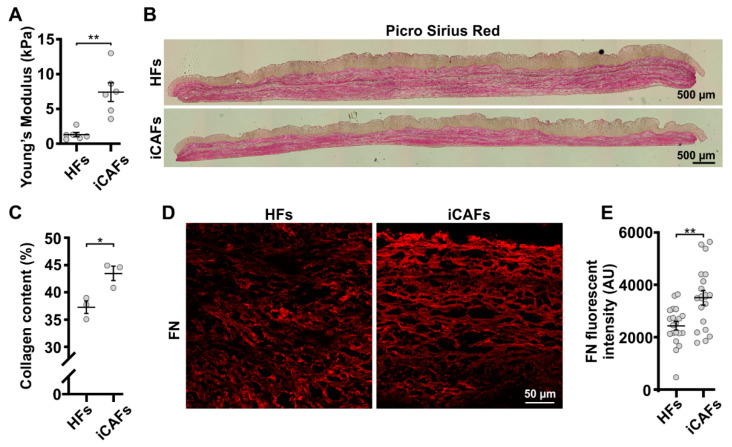
Altered ECM protein content in iCAFs-derived stroma. HFs and iCAFs were cultured in order to produce ECM sheets. Three cell sheets were stacked together, and urothelial cells were seeded on top to create 3D vesical models. (**A**) Young’s elastic modulus computed from tensile deformation test of HFs and iCAFs-derived stroma. N = 6 independent biological replicates. An unpaired *t*-test was performed. (**B**) Tile images of histological sections from HFs and iCAFs-derived models stained with Picrosirius red showing the collagen fibers. (**C**) Corresponding quantification of collagen content (N = 3 independent biological replicates). (**D**) Representative confocal images of HFs-derived and iCAFs-derived constructs stained for fibronectin (FN). (**E**) Corresponding quantification of FN signal based on the background normalized intensity (N = 3 independent biological replicates, 15 images per condition). Data are presented as mean ± SEM. * *p* < 0.05, ** *p* < 0.01.

The tumor stiffening is associated with increased ECM deposition and secretion of matrix remodeling enzymes in the tumor [1,2]. We first asked whether our iCAFs-derived stroma could recapitulate the tumor increased stiffness and associated ECM remodeling. Therefore, we performed tensile deformation tests on the 3D vesical stroma constructs. The elastic modulus for a 5% deformation of the 3D vesical constructs was determined by using a hyper-viscoelastic model to fit the stress–strain curves (Figure S1). Our data showed that the iCAFs-derived stroma was stiffer (E ≃ 7.4 kPa) than the HFs-derived stroma (E ≃ 1.3 kPa) (Figure 1A). Of note, our measured stiffness values closely matched those reported elsewhere for actual bladder tumors [38]. Considering that de novo synthesis of ECM components in tumors, including type I collagen and fibronectin, is attributed to the CAFs [39,40,41,42,43], we next proceeded to characterize the composition of the stroma in the self-assembled 3D vesical tissues when iCAFs were present. To assess collagen content, tissue cryosections were stained for type I and III collagen with Picrosirius red. Notably, the iCAFs-derived ECM had higher collagen content compared to the HFs model (Figure 1B,C). Interestingly, we did not find a significant difference when we compared the mRNA levels of the type I collagen between the HFs and iCAFs cells cultured on 2D plastic (Figure S2A). Using immunostaining, we showed that iCAFs-derived ECM had increased fibronectin expression compared to HFs-derived matrix (Figure 1D,E). Surprisingly, we found lower fibronectin mRNA levels in iCAFs cells cultured on 2D plastic compared to HFs cells (Figure S2B). Overall, we showed that our model can reproduce ECM biochemical switch found in vivo in the tumor microenvironment.

**Figure 2:**
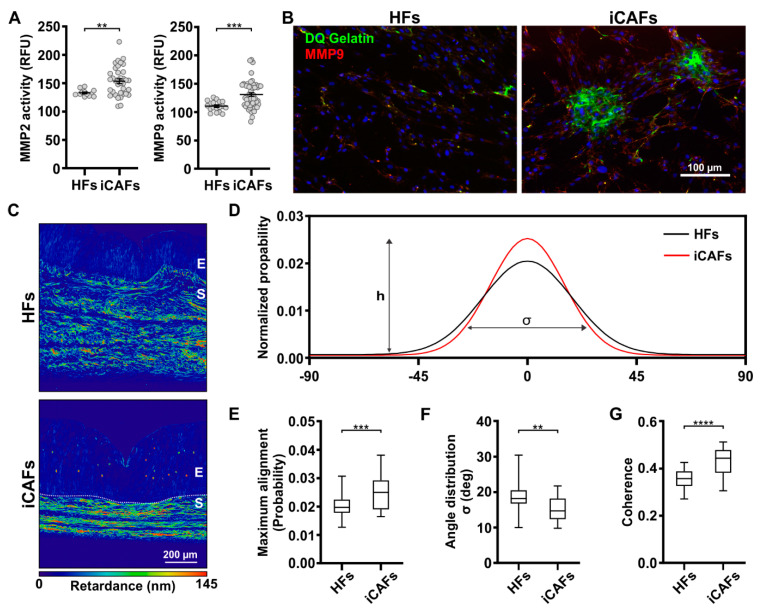
iCAFs-derived 3D vesical model displays increased remodeling of collagen fibers. (**A**) Quantification in relative fluorescence units (RFU) of MMP2 and MMP9 activity secreted by HFs and iCAFs. The data are represented as mean ± SEM. N = 4 independent biological replicates. (**B**) Fluorescence images of the matrix degradation by the HFs and iCAFs visualized with the DQ^TM^ gelatin. The green fluorescence corresponds to gelatin degradation and the red fluorescence to MMP9. Nuclei were counterstained with DAPI. (**C**) Retardance images from HFs-derived and iCAFs-derived constructs stained with Picrosirius red. (**D**) Measured distribution of the collagen fibers’ spatial orientation over a range of 180°, (**E**) along with the corresponding quantification of the peak distribution (h) representing the proportion of collagen fibrils at 0°, (**F**) and the distribution width of the fiber angle (σ). (**G**) Quantification of the coherence of the Picrosirius red signal as a readout of local ECM alignment in the stroma (N = 3 per condition, 30 images per condition). ** *p* ≤ 0.01, *** *p* ≤ 0.001, and **** *p* ≤ 0.0001.

We next investigated whether the induction of the HF cells into CAFs influenced the release of the matrix remodeling enzymes MMP2 and MMP9 metalloproteinases [24,44]. When we assessed the MMPs mRNA expression levels, we found that MMP2 and MMP9 expression was similar between HFs and iCAFs (Figure S2C,D). However, the detected enzymatic activity of the secreted MMP2 and MMP9 was increased in the supernatant from the iCAFs cell culture compared to the HFs (Figure 2A). The iCAFs ability to degrade the ECM was confirmed by a gelatinase assay, which revealed the presence on cleaved fluorescent gelatin around the iCAFs, whereas the signal was mostly absent in HF cells (Figure 2B).

Since altered ECM organization is also a characteristic feature of tumors, we next assessed whether the presence of iCAFs resulted in altered stroma organization and architecture. To evaluate the iCAFs ability to remodel the ECM, we performed a collagen compaction assay. Notably, the measured collagen compaction was greater when iCAFs were seeded in a 3D collagen gel compared to HFs (Figure S3A,B), confirming the greater remodeling abilities of the iCAFs and their greater cellular contractility. We then extended our analysis of the iCAFs remodeling ability in the tumor model by assessing the collagen architecture in Picrosirius stained tissues imaged with the QPOL (Figure 2C) [45]. Notably, we found that the iCAFs-derived collagen was structurally altered, displayed an increased number of collagen fibers aligned along the horizontal axis of the tissue, and presented a tighter distribution of fiber angles compared to the stroma constructs containing HFs (Figure 2D–F). In addition, measurements of the coherency, a quantitative readout of local fiber alignment [46], further confirm the increased fiber alignment in the iCAFs-derived ECM compared to HFs-derived ECM (Figure 2G). Overall, our results indicate that the iCAFs-derived stroma recapitulates the expected physical alteration of a tumor stroma, including the increased stiffness and altered ECM composition and organization.

### 3.2. The iCAFs-Mediated Increased Tissue Stiffness Induces Urothelial Cell Contractility and YAP Nuclear Translocation

**Figure 3:**
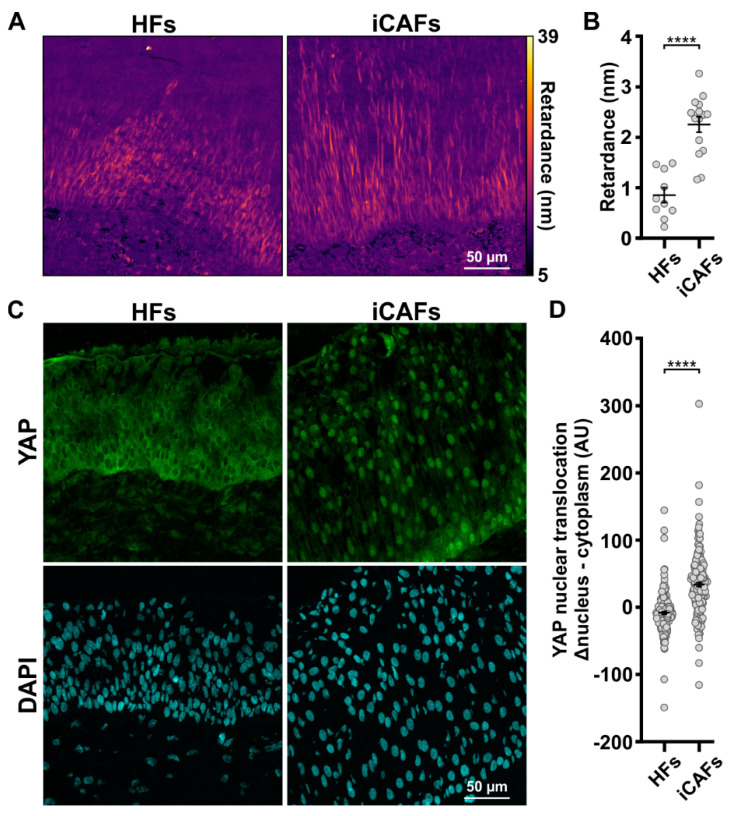
Mechanical forces induced through iCAFs-derived urothelium correlate with YAP nuclear translocation. (**A**) Retardance image of the urothelium on the HFs-derived and iCAFs-derived stroma. Imaging of the optical retardance was performed on label-free cryosections with the QPOL system. (**B**) Corresponding quantification of cell contractility within the urothelium (N = 3 per condition, 30 images per condition). (**C**) Representative confocal images of the urothelial cells seeded on HFs-derived and iCAFs-derived stroma stained for YAP. Nuclei were counterstained with DAPI. (**D**) Corresponding quantification of YAP nuclear translocation by measuring the difference between nuclear expression and cytoplasm expression in urothelial cells. (N = 3 per condition, 15 images per condition). The data are represented as mean ± SEM. **** *p* ≤ 0.0001.

Cell response to increased tissue stiffness involves activating cell contractility and mechanotransduction pathways [47,48]. Therefore, we proceeded to assess the mechanical response of the urothelial cells to the altered ECM in our model by first measuring their contractility levels with the QPOL in stain-free cryosections. For instance, the retardance signal of unlabeled cells is linearly proportional to cell contractility [37]. Specifically, the QPOL retardance signal measured in the urothelium was higher in the iCAFs-derived constructs compared to those on the HFs-derived model, indicative of increased cell contractility (Figure 3A,B). We then proceeded to assess the activation of mechanotransduction by quantifying the nuclear translocation of the mechanosensor protein YAP [49,50]. As expected, we detected higher levels of YAP in the nucleus of the urothelium on the iCAFs-derived constructs compared to those on the HFs-derived constructs (Figure 3C,D). Overall, we determined that ECM-derived mechanical cues affect urothelial cell contractility and mechanoresponses.

### 3.3. iCAFs-Derived Stroma Promotes Proliferation and Infiltration of Urothelial Cells

**Figure 4:**
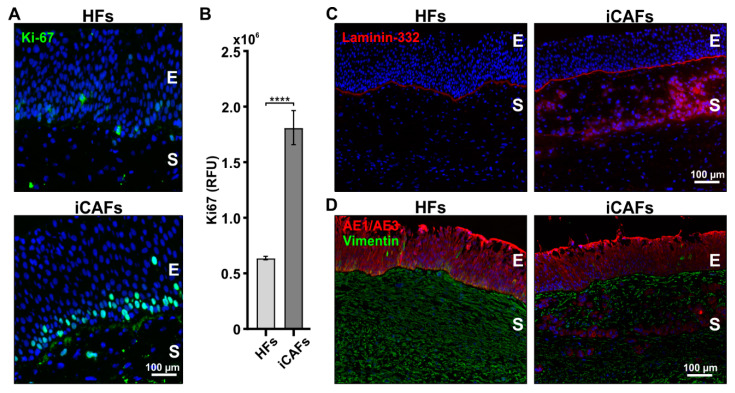
The presence of iCAFs in the stroma of 3D vesical models induces the proliferation and infiltration of urothelial cells. (**A**) Representative fluorescent images of the HFs-derived and iCAFs-derived models stained for Ki67 (green). Nuclei were counterstained with Hoechst (blue). (**B**) Corresponding quantification in relative fluorescence units (RFU) of the Ki67 signal. N = 4 independent biological replicates. (**C**) Representative fluorescent images of the HFs-derived and iCAFs-derived models of laminin-332. (**D**) Representative fluorescent images of vimentin- and epithelium-specific cytokeratin marker AE1/AE3 in the HFs-derived and iCAFs-derived constructs. The images show infiltration of epithelial cells within the stroma in the iCAFs-derived stroma. E = epithelium; S = stroma. The data are represented as mean ± SEM. **** *p* < 0.0001.

The presence of CAFs and an increased matrix stiffness is known to induce several phenotypes, notably increased proliferation and invasion of cancer cells [42,51,52]. We first investigated whether the proliferation ability of urothelial cells was affected by the presence of iCAFs by staining the 3D vesical construct histology sections for the proliferation marker Ki67 [53]. Quantification of the Ki67 fluorescent signal revealed a greater proportion of proliferating urothelial cells in the iCAFs construct compared to HFs constructs (Figure 4A,B). Considering that invasion normally involves the degradation of the basement membrane, we next investigated the organization of the laminin [54]. While all the different constructs displayed what seemed like an intact basement membrane, high levels of laminin-332 were detected into the stromal compartment of the iCAFs-derived constructs (Figure 4C). Given that the laminin staining was positive within the tissue, we proceeded to validate that the urothelial cells were indeed present within the stroma section. To this end, we used AE1/AE3, a cytokeratin marker that specifically labels urothelial cells (Figure 4D). Notably, we observed that urothelial cells infiltrated through the stroma microenvironment in iCAFs constructs while no infiltrating cells were observed within HFs stroma. Together, these results indicate that the presence of iCAFs within the model increased urothelial cell proliferation as well as their infiltration in the stroma.

### 3.4. iCAFs-Derived Models Promote EMT-like State of Urothelial Cells

**Figure 5:**
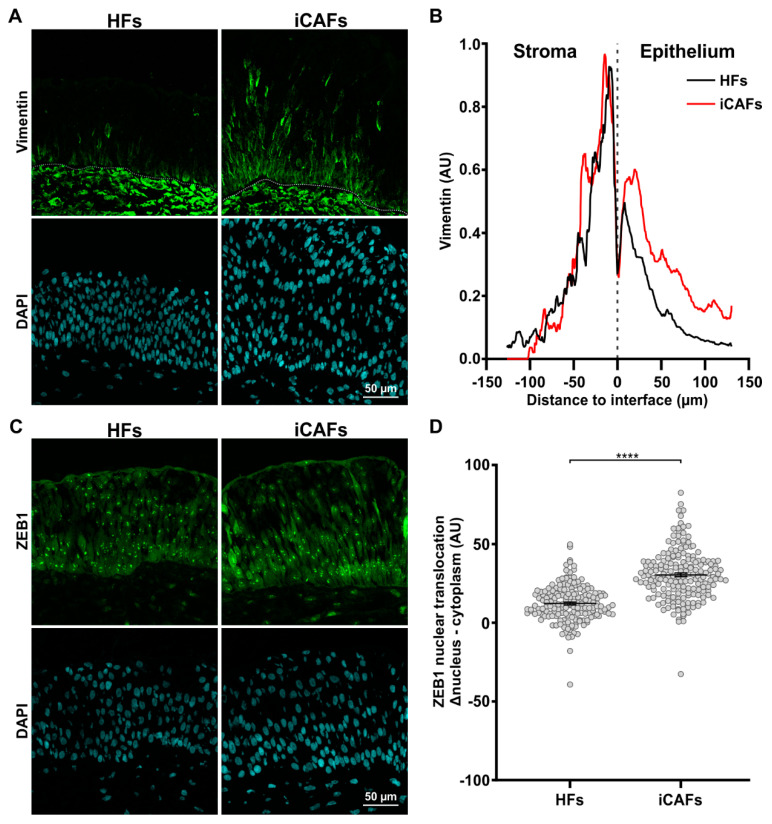
The iCAFs-derived ECM promotes an EMT state in urothelial cells. (**A**) Representative confocal images of the urothelium seeded on HFs-derived and iCAFs-derived stroma and stained for vimentin. (**B**) Corresponding quantification of the vimentin signal across the urothelium. The basal lamina was set as the origin and positive values go toward the top of the urothelium. (**C**) Representative confocal images of the urothelium seeded on HFs-derived and iCAFs-derived stroma and stained for ZEB1. (**D**) Corresponding quantification of ZEB1 signal by measuring the difference between nuclear expression and cytoplasm expression in urothelial cells. Data are presented as scatter plots (N = 3 per condition, 15 images per condition). Nuclei were counterstained with DAPI. The data are represented as mean ± SEM. **** *p* ≤ 0.0001.

Increased ECM stiffness is known to promote and induce the EMT [55]. Given that we observed urothelial cells within the stroma in the presence of iCAFs, we proceeded to investigate if the urothelial cells expressed characteristic EMT markers, namely vimentin intermediate filaments and the transcription factor ZEB1, in the presence of an iCAFs-derived ECM [21,56,57]. Interestingly, we could detect vimentin expressing cells within the basal layer of the urothelium, near the interface with the stroma in the two conditions (Figure 5A). However, quantification of the signal throughout the urothelium revealed that vimentin-expressing urothelial cells extended further away from the interface with the stroma produced by iCAFs compared to those on the stroma found in the HFs (Figure 5B). In addition, we found a proportional increase in ZEB1 nuclear localization in the urothelium on the iCAFs-derived stroma compared to the urothelium on the HFs-derived stroma, with the iCAFs construct displaying the highest ZEB1 nuclear signal (Figure 5C,D). Taken together, and in line with the increased cell contractility, our results indicate that the iCAFs-derived stroma induced expression of at least some mesenchymal markers in the urothelium.

## 4. Discussion

A defining characteristic of tumor progression, including bladder tumors, is the increased matrix deposition and ECM remodeling that results in increased tumor stiffness [58,59]. Several groups, including our own, have established in vitro models trying to capture and engineer at least one of those features [12,60,61,62]. For instance, use of 3D collagen scaffolds can recreate part of the architecture, but achievable stiffness is limited to the lower range of tumor stiffness, even with crosslinking strategies [12,62,63,64]. In the context of bladder cancer, the stiffness of newly diagnosed tumors as measured with atomic force microscopy is around 7.5 kPa, while the stiffness of adjacent non-cancerous tissues is about 2.5 kPa [38]. Interestingly, we were able to achieve a comparable level of tissue stiffness in the construct made from the iCAFs (7.4 kPa) while the HFs-derived stroma was comparatively soft at 1.3 kPa. Our HFs-derived construct remains softer than what was measured for adjacent non-cancerous tissue, likely because the tumor-adjacent tissue in the bladder is not equivalent to healthy tissue [38]. The difference in measured stiffness in our construct is likely due, at least in part, to the increased content in both collagen and fibronectin in the presence of iCAFs. In addition, self-assembled ECM sheets have been shown to be more aligned when they were assembled by activated 3T3 fibroblasts [65]. Similarly, the ECM alignment was increased in our assembled construct in the presence of iCAFs. Moreover, remodeling of the tumor stroma requires the action of different enzymes that are known to be secreted by CAFs, including the MMPs [23]. Indeed, our iCAFs secreted more active MMPs than the HFs, in line with the increased ECM remodeling that was measured in the tissue construct. Overall, we were able to recapitulate the increased tissue stiffness resulting from collagen deposition and ECM remodeling that largely mimics the physical properties found in vivo.

The activation of the mechanotransduction pathways by the increased tumor ECM stiffness plays a major role in regulating cellular processes and behaviors [3,4,66]. As such, the responses of the epithelium in our tumor models are relevant to highlight how the altered mechanical properties of the stroma would eventually influence the urothelium in a patient. For instance, the increase of urothelial cell contractility in the iCAFs constructs matched the increased tissue stiffness, along with a proportional increase in YAP nuclear translocation. Other models from different cancers have shown the same cellular response when altering the stiffness. For example, increased 3D collagen stiffness also results in greater cell contractility of breast cancer cells [37,48]. This mirrors what has been observed in vivo where increased stiffness results in increased cell contractility and activation of mechanotransduction pathways [67]. For bladder cancer, YAP nuclear translocation was also found to be highly correlated with the stromal stiffness [38].

The activation of mechanotransduction pathways can trigger the EMT and is often associated with the upregulation of cellular processes including cell proliferation and invasion [68,69,70]. For instance, YAP has been shown to regulate the expression of mesenchymal markers including ZEB1 and vimentin [71,72,73]. Interestingly, the partial EMT we observed in the urothelium appears to be limited to the cells closer to the interface with the stroma. Based on the profile of both ZEB1 and vimentin, the induced EMT was more robust for the cells on the stiffer tissues, in line with their increased contractility and YAP signal. In addition, the higher tissue stiffness can result in increased cell proliferation in both in vivo and in vitro 3D scaffolds [52,74]. Similarly, we do observe increasing cell proliferation in our models that correlates with the mechanical changes. Interestingly, we observed the presence of urothelial cells within the stroma in iCAFs constructs, clearly indicating that these cells had migrated inside the stroma. However, and even though the cells express EMT markers, the exact mechanism remains unclear since these cells remain nonmalignant urothelial cells. Notably, invasion processes require the degradation of the basement membrane, whereas we could observe laminin around the cells that were inside the stroma [54]. One possibility is that the urothelial cells could have migrated prior to the formation of the basal lamina that normally occurs within the first 7 days of urothelium maturation on the model [54]. Nevertheless, increased ECM stiffness can upregulate 3D migration and could certainly be what drives the cell migration [4,66]. In addition, CAFs have been shown to promote invasion processes through their ability to generate tracks within the stroma or locally align the ECM that invading cells can then take advantage of [52,75,76]. Finally, the CAF secretome could also play a critical role. For instance, we have shown previously that conditioned media from CAFs can induce EMT in non-invasive bladder cancer cells, and promote an invasive phenotype [21]. In addition, conditioned media from CAFs on stiff 3D hydrogel influenced expression of EMT markers in colorectal cancer cells [27]. As such, both the tumor-associated increased ECM stiffness and the CAF secretome could synergistically influence urothelial cells in our model.

Tumor models are designed to get closer to in vivo conditions and increase control over experimental variables such as matrix stiffness and architecture and cellular composition. Extracellular-based 3D hydrogels, including Matrigel and collagen gel are usually more compliant than both normal and tumor tissues [12,62,77,78]. Different approaches can be used to increase the stiffness of 3D hydrogels, including enzymatic (lysyl oxidase and transglutaminase 2), non-enzymatic (glycation), and photoactivable (riboflavin) collagen crosslinkers [79,80,81,82]. Alternatively, synthetic materials such as alginate or polyethylene glycol (PEG) can be used alone or cast into 3D gels to increase stiffness [27,83]. The backbone of these synthetic materials often needs to be modified by introducing metalloprotease-degradable peptides and the cell binding RGD sequences [84]. In contrast, the stiffness of our bioengineered tumor model is close to the range observed in normal and tumor tissues. However, we cannot fine-tune the stiffness of the model with our current approach. Different approaches can be used with 3D hydrogels to control the matrix architecture, including pore size [85,86], introduction of microtracks [87,88] or fibrillar alignment [89]. Our self-assembly model does not permit a direct modulation of ECM architecture. However, we showed an increased collagen alignment and density in our iCAFs-derived model, suggesting that some amount of control over the matrix architecture could be achieved by using different CAF and fibroblast populations. Tumors also contain a number of different cell populations that impact their evolution. For instance, tumor-associated macrophages (TAMs) play a critical role into stimulating fibroblasts’ transdifferentiation and secreting high quantities of TGFβ in the stroma [90,91]. Consequently, other 3D models have included additional cell populations such as immune and endothelial cells [92,93]. While our current model does not recapitulate the cellular heterogeneity of the tumor microenvironment, our approach allows incorporation of multiple cell types. For instance, we have both successfully implanted macrophages and generated a capillary network by embedding endothelial cells in self-assembled tissues [94,95]. Still, our current model is more expensive and lower throughput than other 3D models [93].

Overall, our current iCAFs-derived tissue-engineered models more closely recapitulate the mechanical properties and ECM organization usually reported in tumors. Expanding the scope of our model to include tumor cells, either as organoids or single cells integrated within the urothelium, will provide a powerful platform to investigate tumor progression or response to clinical treatment. However, more precise control on the final mechanical properties of the assembled tissues will be required to fully address the interplay between the CAFs, epithelial and tumor cells. For instance, this study did not address the stiffening of the stroma that occurs through crosslinking enzymes [1]. While adapting the strategy used in vivo to prevent tissue stiffening by targeting these enzymes is likely possible, such an approach will require exhaustive characterization [12]. Finally, by selecting fibroblasts from different organs, or activating them with different tumor cells, the current approach could eventually be adjusted to recreate stroma that better match different kinds of tumor tissues.

Herein, we showed that we can recreate a 3D-tissue-engineered tumor microenvironment with physical properties similar to that of a tumor by using the self-assembly method. We were able to demonstrate the impact of the iCAFs-derived ECM on urothelial cell behavior. Reconstitution of the microenvironment is a major challenge for in vitro models and as such, sophisticated in vitro 3D culture systems are emerging to better recapitulate the microenvironmental aspects of in vivo tumor growth [96,97]. Such improved models are essential to more readily address how the microenvironment drives tumor progression and response to treatment, including the potential interplay between epithelial cells and CAFs. These results established a framework to use CAFs and the 3D self-assembly model for further studies investigating tumor progression and local invasion.

## 5. Conclusions

Here, we showed that we can generate a stroma whose mechanical properties closely match those of tumors in patients by using CAFs in the self-assembly method, and how these changes influence the epithelial cells present on the surface. Notably, our finding indicates that the presence of iCAFs strongly regulates the remodeling of the ECM by enhancing collagen content and fiber alignment. This generalized ECM remodeling leads to the development of a stiffer matrix, which triggers specific biological responses of urothelial cells. Importantly, we also characterized the impact of the engineered tumor stroma on a normal epithelium. We observed that iCAFs-driven mechanical changes upregulate cell contractility and YAP nuclear translocation in urothelial cells. Moreover, we showed that the presence of iCAFs modulates several phenotypes associated with a stiffer tumor stroma, including increased cell proliferation, local cell migration in the stroma, and expression of EMT markers. Overall, our work established how we can leverage and adapt a well-established tissue engineering approach to generate relevant tumor-equivalent stroma and apply them to investigate cellular responses.

## Data Availability

The raw and processed data required to reproduce these findings and the processed results are available on request from the corresponding author.

## Supplementary Materials

The following supporting information can be downloaded at: https://www.mdpi.com/article/10.3390/cancers14153810/s1, Table S1. Primer list. Figure S1. Representative stress-strain curves of HFs-derived and iCAFs-derived ECM constructs during mechanical testing. Figure S2. Impact of tumor cell mediated activation of fibroblasts on ECM and MMPs mRNA expression. Figure S3. iCAFs exhibit increased collagen compaction.
