# Supplementary material for: Cancer-Associated Fibroblasts in a 3D Engineered Tissue Model Induce Tumor-like Matrix Stiffening and EMT Transition
Source: Cancers (Basel). 2022 Aug 5;14(15):3810. doi: 10.3390/cancers14153810 (PMC9367573; doi:10.3390/cancers14153810)
Supplement: Supplementary file 1 [file cancers-14-03810-s001.zip › cancers-1830901-supplementary.pdf]

Supplementary Material

Supplementary Table S1. Primer list

Genes	Primers	Sequences
COL1A1	Forward primer	AGGGACACAGAGGTTTCAGT
	Reverse primer	AGCACCATCATTTCCACGAG
FN1	Forward primer	TCAGCTTCCTGGCACTTCTG
	Reverse primer	TCTTGTCCTACATTCGGCGG
MMP2	Forward primer	CTCTGGACTTAGACCGCTTGG
	Reverse primer	GAAGGTGTTCAGGTATTGCATGTG
MMP9	Forward primer	CGCAGACATCGTCATCCAGT
	Reverse primer	AACCGAGTTGGAACCACGAC

Supplementary figures

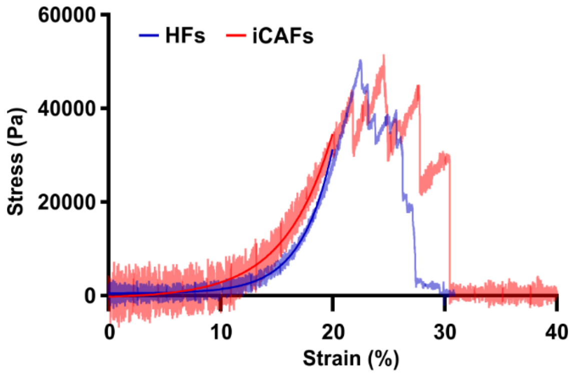

Supplementary Figure S1. Representative stress-strain curves of HFs-derived and iCAFs-derived ECM constructs during mechanical testing. The solid lines represent the calculated fit over the toe region of each sample.

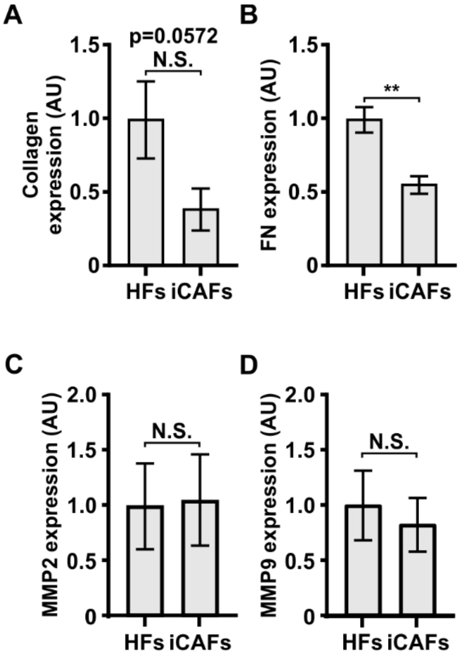

Supplementary Figure S2. Impact of tumor cell mediated activation of fibroblasts on ECM and MMPs mRNA expression. (**A**)**.** RT-qPCR quantification of type I collagen expression in HFs and iCAFs on plastic. (**B**)**.** RT-qPCR quantification of FN expression in HFs and iCAFs on plastic. (**C**). RT-qPCR quantification of MMP2 and (**D**)**.** MMP9 expression in HFs and iCAFs on plastic. Data are presented as mean ± SEM. $^{**}p<0.01$.

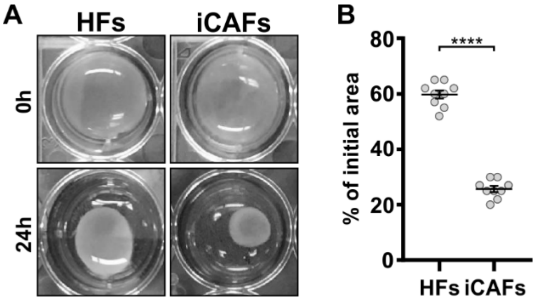

Supplementary Figure S3. iCAFs exhibit increased collagen compaction.

A. Representative images of collagen gel embedded with HFs and iCAFs at 0h and 24h. **B**. Corresponding quantification of the collagen gel compaction after 24h. N=4 independent biological replicates. The data are represented as mean ± SEM. $^{****}p<0.0001$.
